# Antifungal activity of eugenol on *Cryptococcus neoformans* biological activity and *Cxt1p* gene expression

**DOI:** 10.18502/cmm.6.1.2502

**Published:** 2020

**Authors:** Parviz Hassanpour, Masoomeh Shams-Ghahfarokhi, Mehdi Razzaghi-Abyaneh

**Affiliations:** 1 Department of Mycology, Faculty of Medical Sciences, Tarbiat Modares University, Tehran, Iran; 2 Department of Mycology, Pasteur Institute of Iran, Tehran, Iran

**Keywords:** Antifungal effects, Cryptococcus neoformans, Cxt1p gene, Eugenol, Real-time PCR

## Abstract

**Background and Purpose:**

The present study was targeted toward investigating the effects of eugenol on *Cryptococcus* *neoformans* biological activity and *Cxt1p* gene expression.

**Materials and Methods:**

For the purpose of the study, the growth, urease, synergism activity, and disk diffusion of *C. neoformans* were assessed in eugenol-treated culture. The minimum inhibitory concentration (MIC) was determined by the Clinical and Laboratory Standards Institute M27-A3 method at a concentration range of 0.062-2 mg/mL. Subsequently, the expression of *Cxt1p* genes was studied at the MIC_50_ concentration of eugenol using real-time polymerase chain reaction.

**Results:**

The obtained results showed that eugenol at the concentrations of 125 and 500 µg/mL resulted in 50% and 100% growth inhibition in* C. neoformans*, respectively. In terms of urease activity, the results showed that the addition of MIC_50_ of eugenol and fluconazole to urea medium reduced urease activity in *C. neoformans*. In the culture treated with eugenol, the inhibition zone of antifungal drugs, namely amphotericin B, itraconazole, and fluconazole, was increased to 36±0.002, 22±0.001, and 12±0.002 mm, respectively. The expression levels of *Cxt1p* in the eugenol-treated, fluconazole-treated, and non-treated samples were estimated at 46%, 58%, and 100%, respectively.

**Conclusion:**

The findings of the current study revealed that eugenol could cause *C. neoformans* growth inhibition and reduce *Cxt1p* expression in this species. As the results indicated, the susceptibility of *C. neoformans* to fluconazole was increased when combined with eugenol.

## Introduction

*Cryptococcus neoformans* is a relatively frequent agent accounting for serious fungal infections, especially in immunocompromised patients. Based on the evidence, the prevalence of cryptococcal meningoencephalitis in the AIDS patients receiving retroviral drugs is approximately 2% in the United States. However, this rate is almost 30% in South-East Asia and sub-Saharan Africa [1, 2]. *Cryptococcus neoformans* is the main cause of cryptococcosis capable of producing multiple virulent compounds playing a key role in the pathogenicity and host invasion [3]. The main virulence factor of this opportunistic pathogen is its large polysaccharide capsule that surrounds the cell. *Cryptococcus neoformans* strains lacking this capsule are avirulent in animals [4, 5]. Therefore, the synthesis of the capsule could have a therapeutic origin. 

Nearly 97% of the mass of the capsule is made up of two xylose-containing polysaccharides, called glucuronoxylomannan (GXM) and galactoxylomannan (GalXM), and the remaining is mannoproteins [6]. A beta-1,2-xylosyltransferase from *C. neoformans *defines a new family of glycosyltransferases. This enzyme is encoded by the cryptococcal xylosyltransferase (*Cxt1p*) gene, which plays a crucial role in the biosynthesis of the polysaccharide capsule of *C. neoformans*. Animal model studies showed that the elimination of *Cxt1p* results in the reduction of the growth and pathogenicity of this fungus. Several homologs of *Cxt1p* exist in the genome sequence of *C. neoformans* [7]. Systemic fungal infections are mainly caused by the yeasts resistant to such antifungal drugs as fluconazole and itraconazole. 

Azoles and polyenes are the optimal antifungal medications used to treat cryptococcosis with particular limitations because of some side effects and the appearance of drug resistance. The use of natural products originated from plants, such as essential oils (EOs), is another strategy recently administered for the treatment of fungal infections [8-13]. However, in traditional medicine, indigenous populations usually use EOs and plant extracts worldwide [9, 14, 15]. The applied plants contain complex mixtures of volatile (e.g., terpenes, aliphatic aldehydes, alcohols, and esters) and nonvolatile components (e.g., hydrocarbons, fatty acids, sterols, carotenoids, waxes, coumarins, and flavonoids) which are produced by aromatic plants as the secondary metabolites [16-19]. 

Eugenol is the basic constituent of the EO extracted from *Eugenia aromatica*, *Ocimum basilicum*, *Cinnamomum zeylanicum*, and *O. gratissium *[8, 14]. During the last decade, many studies have investigated the pharmacological and therapeutic activities of eugenol, as well as the application of inefficient conventional drugs [14, 20]*.* Such studies have provided potential therapeutic implications for the microorganisms resistant to common antimicrobials [8, 10]*.* With this background in mind, the present study was conducted to investigate the effect of eugenol on *C. neoformans* growth, drug sensitivity pattern, synergism, urease activity, and *Cxt1p* expression using the real-time polymerase chain reaction (PCR) technique.

## Materials and Methods

***Organism, media, and growth conditions***

For the purpose of the study, *C. neoformans* strains PFCC 93-589 were supplied from the Pathogenic Fungi Culture Collection of Pasteur Institute, Iran, (http://fa.pasteur.ac.ir/VisitDetails.aspx?Id=1311) and cultured on Sabouraud dextrose agar (SDA) for 48 h at 37°C. To induce capsule formation, the fungal cells were transferred to the yeast extract-peptone-dextrose (YPD) medium as a capsule-inducing medium (1% w/v yeast extract, 2% w/v peptone, 2% w/v dextrose) at 30C via moderate shaking (150 rpm) [21].

***Testing*** ***s*** ***ubstances and*** ***p*** ***reparation***

Commercial eugenol was purchased from the Sigma Aldrich, E51791 (molecular weight of 164 mg/mL) and kept at 25°C. In addition, a stock solution (6,400 µg/mL) of fluconazole was prepared in dimethyl sulfoxide and stored at -20°C until used.

***Antifungal susceptibility assay***

Antifungal susceptibility assay was conducted according to the guidelines of the National Committee for Clinical Laboratory Standards CLSI M27-A3 method [22]. Briefly, *C. neoformans* was adjusted to 1-5×10^3^ CFU/mL in RPMI-1640 (Sigma-Aldrich, USA), buffered with MOPS medium, and added to a 96-wells plate. The final concentrations of eugenol (0.062-2 mg/mL) and fluconazole (0.5-256 µg/mL) were prepared in RPMI-1640 and added to each well. Subsequently, the plates were incubated at 35°C for 72 h. The minimum inhibitory concentration (MIC) and fungicidal concentration (MFC) of eugenol were determined in the treated samples and compared to those of the fluconazole-treated and non-treated samples. All tests were conducted in triplicate.

***Combined antifungal susceptibility testing by disk diffusion and broth dilution methods***

Combined antifungal susceptibility testing with the disk diffusion method was performed according to the procedure adopted by Pfaller et al. with some modifications [23]. The fungal cell suspension was adjusted to the turbidity of 0.5 McFarland standard and inoculated into Mueller-Hinton agar (Difco Laboratories) containing 2% glucose, methylene blue (0.5 µg/ml), and MIC_50_ of eugenol. Drug disks of fluconazole (FCN; 25 µg), amphotericin B (AMB; 20 µg), flucytosine (10 µg; Mast Diagnostics, UK), and itraconazole (ITR; 50 µg) were applied on the plates. The plates were incubated at 35°C for 48 h; subsequently, the growth inhibition zone was measured. 

The investigation of *C. neoformans* susceptibility to eugenol combined with fluconazole was accomplished using a checkerboard microdilution method, providing a matrix for all possible drug formulations at the required concentration range. The concentration ranges of fluconazole and eugenol were 0.5-256 and 0.062-2 mg/mL, respectively. In addition, 100 mL inoculum suspension was inoculated into flat-bottom 96-well plates containing 50 μL fluconazole and 50 μL eugenol at different concentrations and incubated at 35 C for 72 h [22]. The drug interaction was quantitatively estimated by calculating the fractional inhibitory concentration index (FICI) as follows: 

FICI=(MIC fluconazole combined with eugenol/ MIC fluconazole alone) + (MIC eugenol combined with fluconazole/MIC eugenol alone) 

The drug interactions were categorized as synergism, indifferent, and antagonism if FICI was < 0.5, 0.5-4.0, and > 4.0, respectively. All tests were repeated twice.

***Urease activity assay***

The determination of urease activity was accomplished using the procedure employed by Barbosa Júnior [24]. To this end, a loopful of *C. neoformans* fresh colony was inoculated into the middle of the Christensen agar at a pH of 5.0 (HiMedia, India), containing 20% urea solution, with the MIC_50_ of eugenol and fluconazole and then incubated at 37 C for 72 h [24]. 

***RNA extraction and complementary DNA synthesis***

The MIC_50_ of eugenol and fluconazole was used to evaluate the expression of* Cxt1p* gene. For RNA extraction, the cells were transferred to capsule-inducing media, namely YPD (1% w/v yeast extract, 2% w/v peptone, 2% w/v dextrose) at 30 C with moderate shaking (150 rpm) for 48-72 h. Subsequently, the cells were washed with sterile water at log-phase and then collected. The total RNA was extracted by means of the RNX-Plus Kit, and the quantity and quality of RNA were analyzed using electrophoresis and Nanodrop. According to the manufacturer’s instructions, to avoid any genomic contamination, RNA was treated with *DNase1*. Complementary DNA synthesis was carried out using a kit (Fermentas, USA) following a study performed by Jahanshiri et al. [20].

The primer sequences used in the current study included 5'- CGGAATGGTATGCCTATGTC- 3', 5'-TCTCTTCTCCAGGTTCGCTC-3', 5'- TGC CTCTGGTCGTACCACTG -3', and 5'- GCGAAACCTTCGTAGATGGG -3') for *Cxt1p* and β-actin genes. Real-time PCR was carried out using SYBR green master mix (Applied Biosystems) in a final volume of 20 µl reaction (containing 10 µl real-time PCR 2X Master Mix SYBR, 1 µl of each primer solution [10 mM], 1 µl of total cDNA sample, and distilled water) for each reaction by the ABI PRISM 7500 thermal cycler (Applied Biosystems). The β-actin gene (i.e., an endogenous reference gene) was used for gene normalization. The experiments were repeated in triplicate for each sample. The PCR conditions employed in the current study included an initial incubation at 95°C for 10 min, as well as 40 cycles of 15 sec at 95°C and 1 min at 60°C. Quantitative analysis of the expression level for the investigated genes was carried out using the following formula [20]:

R=2 ^–ΔΔCT^

***Statistical analysis***

All data were analyzed and compared in GraphPad Prism software 6.0 (Sandiego, CA) using the ANOVA test. A p-value less than 0.05 was considered statistically significant.

## Results

***Determination of minimum inhibitory concentration and minimum fungicidal concentration***

The MIC_50_ and MFC values of eugenol were compared with those of the selected antifungal drug (i.e., fluconazole) against the standard clinical isolate of *C. neoformans* (Table 1). The results indicated that 1 (MIC_50_) and 4 μg/mL (MFC) of eugenol resulted in 50% and 100% growth inhibition in *C. neoformans*, respectively.

***Disk diffusion*** ***and antifungal synergy testing***

The results of the disk diffusion test were suggestive of the sensitivity of *C. neoformans* to fluconazole, itraconazole, and amphotericin B. The diameters of the inhibition zone between eugenol-treated and non-treated groups were obtained as 36±0.002, 22±0.001, and 12±0.002 mm for amphotericin B, itraconazole, and fluconazole, respectively. However, flucytosine was not able to inhibit fungal growth (Table 2). 

The FICIs for eugenol combined with fluconazole was also calculated. The results of the checkerboard microtiter assay indicated significant synergistic effects between eugenol and fluconazole against the standard clinical isolate of *C. neoformans* (Table 3). However, the synergistic effects of eugenol and fluconazole against *C. neoformans* showed no significant differences (0.5 > FICI <4.0) after 72 h of incubation at 35°C.

***Urease activity testing***

**Figure 1:**
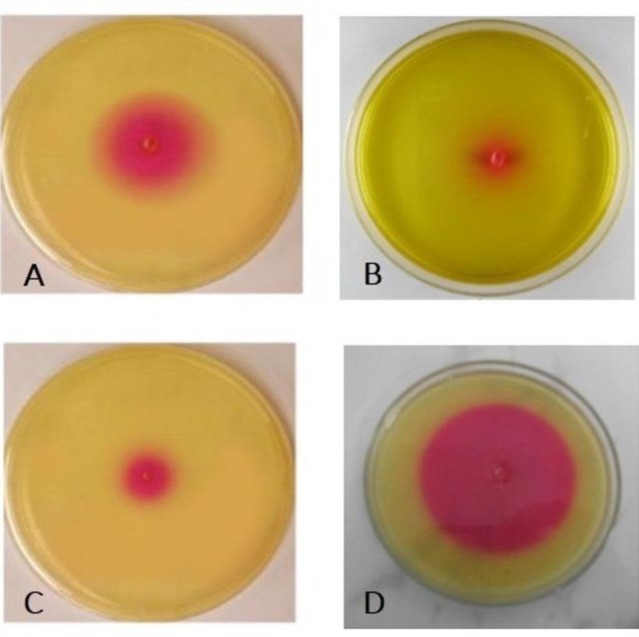
Urea activity of *Cryptococcus* *neoformans;* A) non-treated control, B) eugenol-treated samples, C) fluconazole-treated samples, and D) urease positive-control

Based on the results of growth zone diameter in millimeters, the urease activity test of the yeast showed a significant difference before and after exposure to eugenol and fluconazole at 35°C after 72 h (P<0.05). Furthermore, the measurement of ambient color changes showed urease activity. In this test, the adjacent culture medium with eugenol showed the minimum urease activity (Figure 1).

***RNA extraction, complementary DNA synthesis, and real-time*** ***polymerase chain reaction***

**Figure 2:**
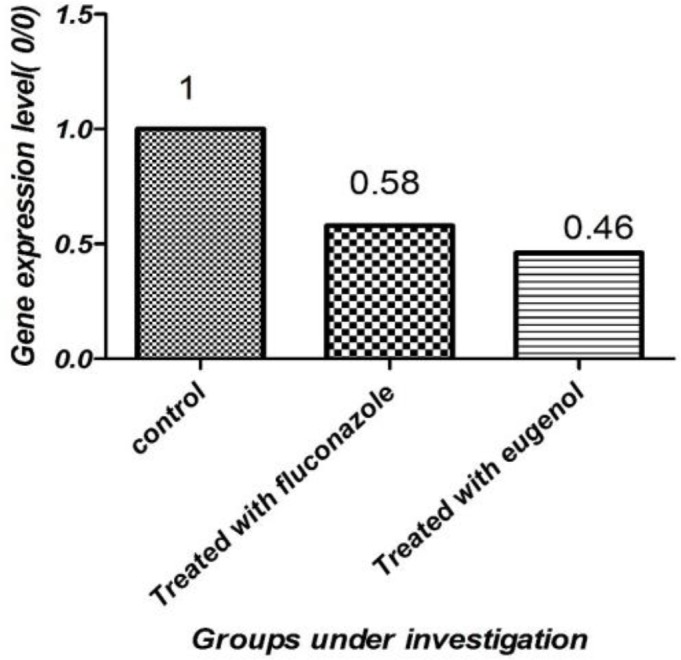
Level of mRNA expression of *Cxt1p* in *Cryptococcus* *neoformans* treated with eugenol (1 μg/mL) and fluconazole (8 μg/mL) (*P<0.05*)

The RNAs extracted from the eugenol-treated, fluconazole-treated, and non-treated specimens were confirmed using electrophoresis and spectrophotometric. After the implementation of cDNA synthesis, a semi- quantitative PCR was performed using specific primers, namely *Cxt1p* and *β-actin*. The results of the real-time PCR technique revealed a significant difference between the eugenol plus fluconazole-treated samples and the non-treated control in terms of *Cxt1p* expression (*P<0.05*). In the mentioned examination, for relative quantitative measurements, the expression of the gene under study (according to the formula $\left[2^{-∆∆\mathrm{CT}}\right]$) in the adjacent fungus with 125 μg/mL (MIC_50_) eugenol and 8 μg/mL (MIC_50_) fluconazole was compared with the gene expression in the control samples. The *Cxt1p* gene expression rates in the eugenol-treated, fluconazole-treated, and non-treated samples were obtained as 46%, 58%, and 100%, respectively (Figure 2).

**Table 1:** Minimum inhibitory concentration and minimum fungicidal concentration of eugenol and fluconazole against *Cryptococcus neoformans* (μg/ml)

**Antifungal compound**	**Mean (range)**	**MIC**_50_	**MIC90**	**MFC**
Eugenol	0.062-2	125	250	500
Fluconazole	0.5-256	8	16	32

**Table 2:** Combination of antifungal susceptibility testing with disk diffusion method (zones of inhibition diameter in mm)

** Antifungal drugs**				
**G** **roups **	**Flucytosine**	**Itraconazole**	**Amphotericin B**	**Fluconazole**
Treated with eugenol	0	22±0.001	36±0.002	12±0.002
Non-treated control	0	20±0.002	30±0.001	10±0.001

**Table 3:** Synergism assay in combination with fluconazole (µg/ml)

**Fungi**	**MIC** **Without combination**		**MIC** **With combination**		**FICI**	**INT**
*C. neoformans*	FCZ	EUG	FCZ	EUG	FCZ+EUG	IND
	8	125	4	3.14	0.75	

FCZ: fluconazole; EUG: eugenol; INT: Interpretation; IND: Indifferent (0.5>FICI<4.0)

## Discussion

Cryptococcosis is a systemic infectious disease mainly caused by *Cryptococcus* genus [4]. The incidence of this infection has increased in the twentieth century due to the emergence of HIV [1]. The administration of common antifungal drugs is not a sufficiently effective measure for the treatment of this disease. The antifungal effects of many organic and natural compounds have been studied against pathogenic fungi [25-34]. According to the literature, eugenol has antifungal activities against *C. neoformans*, *C. albicans,* and *A. parasiticus* [20, 32, 35-38]. 

The present study involved the investigation of the efficacy of eugenol in *C. neoformans* growth, urease activity, antifungal susceptibility, and *Cxt1p* gene expression. At the first stage of our study, the effect of eugenol was evaluated on* C. neoformans* growth. The results showed that eugenol at a concentration of 125 μg/mL declined the fungal growth rate by 50%, while at the concentration of 500 μg/ml, it completely inhibited fungal growth. In a similar study, Alves et al. examined the antifungal and antioxidant activities of eugenol against *C. neoformans* strains. In the mentioned study, the mean MIC_50_ of eugenol was determined as 256 mg/L, and it was shown to completely inhibit fungal growth at higher concentrations [35]. 

In another study, the antifungal and anti-biofilm activities of the active ingredients of EOs were evaluated against* C. neoformans* and *C. laurentii*. The results of the mentioned research revealed the higher susceptibility of *C. neoformans* and *C. laurentii* to polyene medications (e.g., amphotericin B and nystatin) than to fluconazole (32 and 16 μg/mL). The MIC_80_ values of thymol, carvacrol, citral, eugenol, and menthol against *C. neoformans* were measured at 32, 16, 64, 128, and 64 μg/mL, respectively [36]. Another study involved the investigation of the antifungal activities of eugenol and clove plants against *C. neoformans* and *Candida albicans.* In the mentioned research, the MIC values of eugenol and clove plants for *C. neoformans* were determined as 6.28 and 2.43 mg/mL, respectively [37]. In addition, the antifungal activity of *Osmium gratismum* containing eugenol (16%) was tested against *C. neoformans,* and the results showed that fungal growth was inhibited at a concentration of 0.9 μg/ml [8]. 

Combination therapy with antimicrobial herbal extract can prevent the side effects and resistance mechanism of antifungal drugs [10, 11, 29]. In our study, after the combination of eugenol with fluconazole, eugenol showed no difference in *C. neoformans *growth inhibition. In a study, the antifungal activity of eugenol and methyleugenol alone and in combination with fluconazole was indicated against clinical *Candida* isolates. In the mentioned research, the FICI values depicted a high synergism of fluconazole with both compounds, which was at the highest value with methyleugenol (FICI=0.2-0.5). Furthermore, fluconazole-resistant *Candida* isolates showed a high sensitivity to both compounds [38]. 

Alves et al. observed no interaction between eugenol and such antifungal drugs as fluconazole and amphotericin B against *C. gattii* and *C. neoformans* [38]. In the current study, the synergistic effect of eugenol was shown at a concentration of 125 μg/mL. In this regard, eugenol greatly increased the sensitivity of* C. neoformans *to amphotericin B, itraconazole, and fluconazole. However, the samples treated with eugenol showed no significant increase in susceptibility to flucytosine. It seems that the susceptibility of *C. neoformans* to antifungal drugs, such as fluconazole, increases when combined with eugenol. 

The investigation of urease activity in *C. neoformans* showed that the addition of eugenol (125 µg/mL) and fluconazole (8 µg/mL) to the urea medium culture decreased the urease activity of* C. neoformans*, compared to that in the controls. The inhibition zone diameters of eugenol-treated, fluconazole-treated, and non-treated control groups were measured at 10, 20, and 30 mm, respectively. In a study addressing the activity of urease *C. neoformans*, Barbosa Júnior et al. reported that the level of urease activity in the environmental and clinical isolates of *C. neoformans* was negligible after 24 and 48 h; however, the level of urea production was significant after 7 days [24]. In another investigation, Liaw et al. reported the occurrence of a moderate urease activity (37.5%) in *C. neoformans* special complex isolates obtained from Taiwan after 48 h and a low level of urease production (62.5%) [39]. 

The* Cxtp1* expression in *C. neoformans* in the samples treated with eugenol and fluconazole underwent a decrease in comparison with that in the non-treated controls. In the current study, 46% eugenol and 58% fluconazole reduced the expression of *Cxt1p*. Klutts et al., investigating the role of *Cxt1p* in the biosynthesis of *C. neoformans* capsule, reported that the loss of *Cxt1* did not affect the growth and general morphology of their capsules in the mutant cells in vitro; however, the two main capsular polysaccharides, namely GXM and GalXM, were missing beta1,2-xylose residues, compared with wild-type strains [7]. 

In other studies, eugenol was reported to affect the expression of regulatory genes in fungal metabolic pathways. Jahanshiri et al. observed that eugenol strongly inhibited *Aspergillus parasiticus* growth within a range of 19.16-95.83% in a dose-dependent manner. Aflatoxin B1 production was also inhibited by the compound within a range of 15.07-98.0%. In addition, the expression of *ver-1*,* nor-1*, *pksA*, *omtA,* and *aflR* genes were also found to be significantly suppressed by eugenol at the concentrations of 62.5 and 125 μg/mL [20]. Yörük et al. reported significant differences in fold changes in gene expression in *Fusarium*. In the mentioned study, fold changes in *FcMgv1 *and *FcStuA *genes were measured at +4.35±0.25 and +2.04±0.13, respectively, based on the normalization results [40]. The results obtained from the aforementioned studies on the antifungal effect of eugenol are indicative of the ability of this compound to modify fungal growth and metabolically-related gene expression; therefore, it can be used as a potent inhibitor for therapeutic purposes.

## Conclusion

As the results of the present study indicated, eugenol is an effective factor in *C. neoformans* growth and *Cxt1p* expression (i.e., an effective gene in capsules biosynthesis and pathogenicity). I the present study, eugenol facilitated the enhancement of the susceptibility of *C. neoformans* resistant to fluconazole, compared to that to amphotericin B. Our findings were indicative of the increased susceptibility of *C. neoformans* to fluconazole when combined with eugenol. It is required to perform further studies with fluconazole-resistant strains in order to confirm this hypothesis.
